# Development, Reliability, and Validity Assessment of a Portable 3D Camera-Based System for Quantifying Postural Sway and Balance

**DOI:** 10.3390/s26133987

**Published:** 2026-06-23

**Authors:** Vivek Ganesh Sonar, Vibhor Agrawal, Krushal Kalkani, Javad Hashemi, Abhijit Pandya

**Affiliations:** 1 Department of Computer Science & Electrical Engineering, Florida Atlantic University, Boca Raton, FL 33431, USA; pandya@fau.edu; 2 Department of Population Health, College of Medicine, Florida Atlantic University, Boca Raton, FL 33431, USA; agrawalv@health.fau.edu (V.A.); kkalkani2024@fau.edu (K.K.); 3 Department of Biomedical Engineering, Florida Atlantic University, Boca Raton, FL 33431, USA; jhashemi@fau.edu

**Keywords:** postural sway, 3D depth camera, center of mass, reliability, validity

## Abstract

Accurate assessment of postural sway is essential for evaluating balance disorders, rehabilitation outcomes, and fall risk. Traditional laboratory-based motion capture systems provide precise center-of-pressure (CoP) measurements, but are expensive, non-portable, and impractical for widespread clinical use. This study describes the development and testing (reliability and validity) of a portable three-dimensional (3D) camera system (Intel RealSense D415) for quantifying sway and balance. Test–retest reliability was evaluated in healthy adults (n = 10; 6 males, 4 females; mean age 22.3 ± 1.6 years), yielding intraclass correlation coefficients ICC = 0.84–0.86 (95% CI: 0.61–0.95). Concurrent validity, established against a laboratory-based optical motion capture system (Optotrak), demonstrated strong correlations with a mean absolute percentage error of 10.5% relative to Optotrak-derived path length measurements and high levels of agreement. Operating at 30 Hz with end-to-end latency of <40 ms, the RealSense-based system provides a reliable, valid, and portable alternative to lab-based systems. Low-cost markerless motion capture systems based on standard RGB cameras have been validated for postural risk assessment, showing good consistency with gold-standard Vicon systems. These preliminary findings suggest that the system shows promise as a low-cost alternative; however, further validation in clinical populations is required before clinical deployment.

## 1. Introduction and Background

Balance control is a fundamental human function that depends on the continuous integration of sensory inputs from the visual, vestibular, and somatosensory systems and produces appropriate motor responses. Disturbances in the sensory and/or motor processes manifest as increased postural sway. Sway has emerged as a sensitive biomarker for neurological and musculoskeletal disorders, such as Parkinson’s disease, and for fall risk [1,2,3]. Falls remain one of the leading causes of injury and mortality in older adults, with an estimated one in three individuals over the age of 65 experiencing at least one fall each year [2]. Accurate and repeatable measurement of sway is therefore a clinical priority in the areas of geriatrics, neurology, rehabilitation, and sports medicine.

Falls represent a major global public health concern, particularly among older adults, and are the second leading cause of unintentional injury deaths worldwide [2]. The growing aging population has intensified research in wearable and camera-based technologies for fall detection and balance monitoring over the past decade [4].

In parallel, markerless motion capture systems using video cameras and depth sensors have emerged as non-intrusive tools for quantifying human movement and balance. Such systems have been applied for fall risk assessment using machine learning approaches [5] and for monitoring functional performance in clinical populations with neurodegenerative conditions [6], highlighting the growing potential of camera-based systems for accessible, real-world balance evaluation. Depth camera systems such as the Microsoft Azure Kinect have demonstrated strong accuracy and repeatability for clinical motor function assessment [7], and multi-camera depth sensor setups have further improved tracking reliability by reducing occlusion errors [8]. Vision-based 3D pose tracking using RGB–depth sensors has been applied to assess postural instability in Parkinson’s disease, demonstrating excellent agreement with gold-standard marker-based motion capture [9]. Computer vision-based gait analysis using pose estimation has been applied to unobtrusively assess mobility and fall risk in older adults with dementia in real-world environments [10]. A systematic review of 65 studies confirmed that markerless motion capture technology is increasingly applied for clinical measurement in rehabilitation, with Parkinson’s disease patients representing the largest group studied [11].

Traditional methods of quantifying sway rely on laboratory-grade instruments such as force platforms, which measure ground reaction forces, and optical motion capture systems that compute center-of-mass (CoM) trajectories. While these instruments are considered the gold standard, they are expensive, immobile, and require trained personnel, restricting their use to specialized laboratories. Such limitations severely hinder their widespread clinical adoption, particularly in primary care, home-based rehabilitation, and tele-health environments. Alternative solutions such as inertial measurement units (IMUs), pressure mats, and low-cost gaming devices (e.g., Wii Balance Board, Kinect) have been investigated, but they often lack sufficient accuracy, robustness, or three-dimensional fidelity for clinical decision-making [12,13]. Inertial measurement units (IMUs) have been widely explored as portable alternatives, demonstrating acceptable reliability and validity for fall risk assessment in community-dwelling older adults [14]. Systematic reviews have confirmed that IMU-based sensors provide moderate-to-good reliability for both static and dynamic balance in healthy adults [15].

Recent advances in computer vision and depth-sensing technologies have introduced new opportunities for markerless and portable balance assessment. A scoping review of single-camera markerless motion capture confirmed its expanding role in healthcare applications, highlighting strong potential for clinical balance and movement assessment [16]. The Intel RealSense D415, a stereoscopic depth camera with global shutter technology and millimeter-level precision at close range, enables the acquisition of three-dimensional (3D) skeletal data without markers or specialized equipment [17,18]. When combined with modern pose estimation frameworks, such as the MediaPipe Pose—which tracks 33 anatomical landmarks in real time—this technology can provide a complete representation of whole-body postural control [19]. By applying biomechanical models (e.g., Zatsiorsky–Seluyanov segmental mass distribution with de Leva’s adjustments), the body’s center of mass (CoM) can be estimated, projected to the ground plane, and analyzed to derive clinically relevant stabilogram features such as path length, mean velocity, sway ellipse area, and directional root mean square (RMS) sway [20].

It is important to note that force platforms measure the center of pressure (CoP)—the point of application of the ground reaction force—whereas the proposed system estimates the projected center of mass (CoM), the mass-weighted average position of the body segments. Although CoP and CoM trajectories are closely related during quiet standing, they are not biomechanically equivalent, and this distinction is maintained throughout the manuscript.

Recent studies have explored portable and wearable alternatives for balance assessment outside of laboratory settings. Inertial measurement units (IMUs) have shown promising results as low-cost tools for measuring postural sway, demonstrating feasibility in elderly populations [21,22] and acceptable reliability and validity for fall risk assessment in community-dwelling older adults [14]. Furthermore, wearable IMU sensors have demonstrated construct validity in measuring postural sway under varying visual conditions [23], supporting their use as practical alternatives to traditional force plate systems.

The potential of 3D cameras for clinical balance evaluation lies in their affordability (approximately USD 200), portability, and non-intrusive, markerless operation. Unlike two-dimensional video or single-sensor systems, depth cameras capture anterior–posterior and mediolateral sway simultaneously, as well as trunk inclination, allowing for a more comprehensive assessment of postural control. Furthermore, their ease of deployment and ability to operate in real time make them suitable for diverse environments, including outpatient clinics, rehabilitation centers, community health programs, and even remote home monitoring [24,25]. Smart wearable and camera-based technologies for balance rehabilitation have been identified as promising solutions for the growing falls epidemic in older adults, offering objective movement data, real-time feedback, and accessibility beyond clinical settings [25]. Therefore, this study aimed to determine whether a low-cost, markerless 3D camera system can serve as a reliable alternative to traditional laboratory systems for balance assessment in clinical and remote care settings.

### Study Objectives

The primary objectives of this study were (1) to develop a software program for quantifying postural sway in human subjects using the Intel RealSense D415 3D camera model, and (2) to rigorously evaluate the reliability and validity of this system. Specifically, this research pursued four sub-objectives. First, system development and algorithm integration: to design and implement a real-time processing pipeline that acquires synchronized RGB–depth data, applies skeletal pose estimation, reconstructs 3D joint trajectories, and computes whole-body CoM dynamics using biomechanical models to deliver clinically interpretable stabilogram features with minimal latency. Second, test–retest reliability: to quantify the inter-session consistency of path lengths in healthy adults under standardized sway tasks. Third, concurrent validity: to evaluate the degree of accuracy between RealSense-derived sway measures and those obtained simultaneously from a laboratory-based optical motion capture system. Fourth, to evaluate the system’s practical feasibility for deployment in non-laboratory settings by assessing its portability, setup time, and ease of use by a non-specialist operator.

Through these objectives, this study seeks not only to validate the RealSense-based 3D system as a clinical instrument but also to establish its practical role in accessible, scalable, and reliable balance assessment across diverse healthcare environments. This project was performed in two phases to address each study objective: Phase I—Development of the software program, and Phase II—Experimental evaluation.

## 2. Methodology

Phase I—System Development: The proposed system was designed to overcome the shortcomings of the current sway assessment technology and to meet the needs of clinicians.

Current Technologies for Sway Assessment:

Postural sway assessment has been extensively studied using force platforms, motion capture systems, inertial sensors, and, more recently, markerless vision-based technologies. RGB–depth camera systems have been used to establish normative data for standardized motor tasks, demonstrating their suitability for clinical assessment beyond the laboratory [26]. Accelerometer-based postural sway assessment has been directly compared against force plate measures, confirming that portable inertial sensors provide a viable and accessible alternative to laboratory equipment [27]. Force platforms remain the gold standard, offering precise center-of-pressure (CoP) trajectories with high temporal resolution and clinical reliability [1,28,29]. Recent studies have also confirmed the feasibility of wearable IMU sensors for elderly postural sway assessment [21], with construct validity demonstrated under varying visual conditions [23]. Sensor-based interventions have shown significant improvements in gait and balance performance in older adults in randomized controlled trials [30]. However, their high cost, immobility, and requirement for controlled laboratory conditions limit their deployment in broader clinical and community settings [28,31]. Optical motion capture (Mocap) systems such as Vicon and OptiTrack have also been employed to estimate sway by reconstructing full-body kinematics with millimeter accuracy. While highly precise, these systems are labor-intensive, require reflective markers, and are restricted to specialized laboratory environments [28,31].

To enhance portability, inertial measurement units (IMUs) have been explored for balance monitoring. Wearable IMUs capture linear accelerations and angular velocities of body segments and have shown moderate-to-good reliability for sway metrics (intraclass correlation coefficient (ICC) ≈ 0.57–0.79) [13]. Despite their usefulness in ambulatory monitoring, IMUs provide only indirect estimates of CoM movement and are sensitive to sensor placement and drift. Other low-cost alternatives, such as the Wii Balance Board and the BTrackS Balance Plate, have been used for screening purposes. These force-based devices offer acceptable reliability in certain contexts, but lack kinematic data and often demonstrate reduced sensitivity compared to laboratory force plates [32]. Wearable plantar pressure sensors combined with machine learning have also demonstrated promise for fall risk detection in community settings, offering interpretable biomechanical markers such as pressure duration, intensity, and symmetry [33].

Unlike two-dimensional video or visual observation, 3D approaches capture sway in multiple planes—anteroposterior (AP), mediolateral (ML), and vertical—as well as trunk inclination, and provide a more comprehensive assessment of postural control. They thus overcome many of the limitations of visual estimation and traditional instruments by providing objective, markerless, and real-time quantification of body movement. Earlier work with the Microsoft Kinect demonstrated promising correlations with force plate data (r ≈ 0.82–0.90) during quiet standing [34,35,36]. However, accuracy limitations such as occlusion sensitivity and depth distortion restricted its adoption for clinical practice [12,35].

By contrast, newer RealSense devices offer improved spatial resolution, sub-millimeter depth fidelity, and robust skeletal tracking capabilities [17,18]. The RealSense D415 and D435 models can generate synchronized RGB and depth streams that enable precise localization of anatomical landmarks and estimation of the body’s CoM [17,18]. RGB-D sensors have also been validated for sitting balance assessment, with a body angle measurement agreement of 2.19 ± 2.29 degrees compared to reference measurements [37]. Integration of OpenPose with RGB-D cameras has provided an effective method for identifying 3D body landmark locations across various postures, supporting ergonomic and postural assessment applications [38]. Depth cameras have been shown to be a feasible and affordable alternative to force plates for tracking center of mass during standing balance training, demonstrating Pearson’s correlation above 0.98 compared to gold-standard force plate measurements [39]. Waist-mounted triaxial accelerometers have been used to predict Short Form Berg Balance Scale scores in older adults during Timed Up and Go tests, confirming the feasibility of low-cost wearable devices for functional balance screening [40]. RGB–depth camera systems have further been validated as non-radiographic, low-cost alternatives to traditional gold-standard postural analysis tools [41,42], supporting their use in both clinical and community-based settings. The findings of this study are consistent with the growing body of literature demonstrating that portable devices are effective for balance and postural sway assessment. When paired with frameworks such as MediaPipe Pose [19], they can provide accurate 3D joint reconstruction in real time [43]. Emerging applications of RealSense have included upper-limb kinematics [24] and thoracic spine posture monitoring [44], underscoring its versatility in motion analysis. Nonetheless, systematic evaluation of its reliability and validity for whole-body sway quantification remains limited, leaving an important opportunity for research. Given their affordability, portability, and clinical precision, RealSense-based 3D systems have strong potential for widespread integration into rehabilitation, tele-health, and community-based balance assessments.

Current Clinical Practices for Sway Assessment:

In many clinical environments, balance and postural stability are often assessed informally through visual observation by physicians, therapists, or nurses. Although experienced clinicians can recognize gross abnormalities in balance, such assessments are inherently subjective and highly dependent on the observer’s expertise. Inter-rater variability is common, and subtle sway differences on the order of only a few millimeters are almost impossible to detect without instrumentation. As a result, visual estimation lacks the sensitivity required for early detection of balance impairments and for monitoring incremental improvements during rehabilitation. Furthermore, visual assessments cannot generate quantitative metrics such as path length, sway velocity, or ellipse area, which are critical for objective documentation and clinical decision-making.

The reliance on subjective judgment reduces reproducibility and limits the integration of balance testing into standardized diagnostic protocols. Consequently, there is a clear need for objective, quantitative tools—such as 3D camera-based systems—that can complement clinical expertise with precise, repeatable measurements. Attention-based deep learning models combined with wearable IMU sensors have been proposed to automate Berg Balance Scale scoring, reducing dependence on trained clinicians and improving scalability [45]. A separate deep learning framework validated on 4800 samples further confirmed the feasibility of automated balance evaluation using wearable IMU sensors for neurological conditions such as stroke and Parkinson’s disease [46]. The proposed software design addresses these needs for establishing a clinically robust, generalizable, and scalable balance assessment tool.

A. Design goals and acceptance criteria

- Primary output: Stabilogram of whole-body CoM projected on the ground plane and derived sway metrics (path length (PL), mean velocity (MV), root mean square (RMS) in anterior–posterior (AP) and medial–lateral (ML) directions, ellipse area (EA), and trunk angle (θ)).
- Throughput target: ≥30 FPS end-to-end (capture → pose → CoM → features → GUI).
- Latency budget: <100 ms pipeline latency (capture to on-screen metrics), split roughly as: capture (10–20 ms) + pose inference (15–35 ms) + depth fusion (5–10 ms) + filters and metrics (5–15 ms) + rendering (5–10 ms).
- Reliability hooks: Missing-data handling, artifact detection, QC gates (coverage, dropouts, tracking loss).
- Portability: Single Intel^®^ RealSense™ D415 + laptop; no markers, no force plate required for runtime.

B. Hardware, optics and physical setup

- The system uses an Intel RealSense D415 RGB-D camera (Intel Corporation, Santa Clara, CA, USA) with 16-bit depth at 640 × 480 resolution, operating at 30 Hz with the depth stream aligned to the color stream. The camera is mounted on a tripod at 1.1–1.2 m in height, positioned 2.0 m from the subject with approximately 5° downward pitch to keep both feet fully in the field of view. Uniform diffuse lighting is used, and strong infrared sources are avoided. A 2 s pre-roll period allows auto-exposure and gain stabilization before each trial. The system runs on a Lenovo Legion 7 laptop with a CUDA-capable GPU, maintained below 80% utilization to prevent thermal throttling. The camera is configured with the High Accuracy depth preset, emitter power of 150–330 mA, auto-exposure enabled, depth-to-RGB alignment enabled, and a frame queue size of 3–5 to minimize latency while avoiding frame drops. Single-camera markerless motion capture has been identified as a practical and accessible approach for healthcare movement analysis, with demonstrated applications across rehabilitation, fall risk screening, and postural assessment [16].

C. Software stack and modular architecture

- The software architecture consists of several modular components: the RealSense SDK handles synchronized RGB and depth capture at 30 Hz; MediaPipe Pose performs skeletal landmark detection (33 landmarks) on the RGB stream, with depth values sampled per-landmark from the aligned depth map; camera intrinsics are used for back-projection, with the world frame fixed to the ground plane; biomechanical modeling follows the Zatsiorsky–Seluyanov segment mass model with de Leva adjustments for whole-body CoM estimation; signal processing includes interpolation, Butterworth low-pass filtering, and Hampel outlier suppression; a real-time four-panel user interface displays the RGB stream with skeletal overlay, a depth heat map, a stabilized 3D stick figure, and live metrics; and per-frame data including timestamps, 3D joint positions, CoM coordinates, and all derived metrics are logged to CSV. MediaPipe-based markerless tracking has been evaluated against gold-standard systems for 2D trajectory accuracy, demonstrating promise as a lightweight AI-based motion capture solution for clinical settings [47].

D. Acquisition and timestamping (RGB-D)

D.1 Stream init

1. Open RGB stream (640 × 480, 30 Hz), Depth stream (640 × 480, 30 Hz), aligned.
2. Warm-up: discard first 60 frames (~2 s) for AE/gain stabilization (the pre-roll period described in Section B).
3. Pre-allocate ring buffers (length 90–120 frames) for RGB, Depth, and Landmarks.

D.2 Frame loop (pseudo)

while running:

rgb, depth, ts = camera.next_frame() # ts = hardware timestamp (ms)

rgb_bgr = cvtColor(rgb, BGR)

enqueue(rgb_bgr, depth, ts)

D.3 Timebase

- Use RealSense hardware timestamps as the primary clock. Store t_capture, and later annotate t_pose_done, t_metrics_done for latency profiling.

E. Pose estimation (RGB → 2D landmarks)

E.1 Preprocess

- Resize RGB to model input [e.g., 256–640 px shortest side], keep aspect ratio; normalize to [0, 1].

E.2 Inference

MediaPipe Pose (full-body) produces 33 anatomical landmarks in 2D image coordinates, each with a per-landmark visibility score $v_{j}\in \left[0,1\right]$, representing the model’s confidence that the joint j is correctly detected. The model also outputs a global pose detection score $s\in \left[0,1\right]$, which reflects the overall confidence that a valid human pose is present in the frame. In this study, frames with s<0.5 were rejected from further processing; all results reported in this paper use this fixed threshold.

Definition—local depth dropout rate. For each landmark j at pixel location $\left(u_{j},v_{j}\right)$, a 3×3 pixel neighborhood $N_{j}$ is sampled from the aligned depth map. Let $n_{\mathrm{invalid}}$ denote the count of pixels in $N_{j}$ that return zero, fall outside the valid range $\left[z_{\min},z_{\max}\right]$, or are flagged by the sensor as unreliable. The local depth dropout rate for the landmark j is defined as:

(1) $$D_{j}\frac{n_{\mathrm{invalid}}}{|N_{j}|=\frac{n_{\mathrm{invalid}}}{9}}$$

Here *n_invalid_* is a single count (a labeled variable), if $D_{j}>0.20$ (i.e., more than 20% of the neighborhood pixels are *n_invalid_*), the depth reading at that landmark is marked as missing for that frame and handled by the temporal interpolation procedure described in Section H. The terms “local dropout” and “depth dropout” used elsewhere in this paper refer to this same quantity $D_{j}$.

E.3 Landmark smoothing (2D)

Exponential moving average (EMA). To reduce frame-to-frame jitter in the 2D landmark coordinates prior to depth sampling, an exponential moving average filter is applied independently to each landmark’s horizontal and vertical pixel coordinates. For a landmark coordinate p (either u or v), the smoothed value at frame t is computed as shown in Equation (2), where α is the smoothing factor controlling the weight given to the current versus previous framed as:

(2) $${\hat{p}}_{t}=\alpha \cdot p_{t}+\left(1-\alpha \right)\cdot {\hat{p}}_{t-1}$$

where $p_{t}$ is the raw coordinate output by MediaPipe at frame t, ${\hat{p}}_{t-1}$ is the smoothed value from the previous frame, and $\alpha \in \left(0,\;1\right]$ is the smoothing factor. A higher α gives more weight to the current observation (less smoothing), while a lower α produces heavier smoothing at the cost of increased lag. In this study, α=0.35 was used, selected empirically to balance jitter suppression with responsiveness at 30 Hz. Values of α between 0.2 and 0.5 were tested; α = 0.35 produced the best balance between noise reduction (RMS jitter reduced by ~60%) and lag (mean delay < 1 frame at 30 Hz), and was therefore adopted for all analyses.

F. Depth fusion at landmarks (2D → depth z)

F.1 Sampling window

Depth sampling at landmark locations. For each of the 33 landmarks, the pose estimator provides a 2D image coordinate $\left(u_{j},v_{j}\right)$ in the color frame. Because the depth map is aligned to the color frame, the depth value at that pixel can be read directly. However, individual depth pixels from stereo cameras are subject to speckle noise (random high-frequency depth fluctuations caused by interference patterns in the infrared projector). To mitigate this, a two-step spatial filtering procedure is applied within the 3×3 pixel neighborhood $N_{j}$, centered at $\left(u_{j},v_{j}\right)$:

1. Median filter. The nine depth values in $N_{j}$ are sorted, and the median value is selected. The median filter suppresses isolated outlier pixels (speckle) without shifting the central depth estimate, and its kernel size is fixed at 3×3 pixels throughout this study.
2. Bilateral weighting. To prevent depth averaging across object boundaries (e.g., the edge between a limb and the background), a bilateral weight is applied. Each neighbor pixel $k\in N_{j}$ receives weight:

(3) $$w_{k}=\exp\left(-\frac{{\left(d_{k}-d_{med}\right)}^{2}}{2\sigma_{d}^{2}}\right)$$

where $d_{k}$ is the depth at the neighbor pixel k, $d_{\mathrm{med}}$ is the median depth from Step 1, and $\sigma_{d}$ controls the range tolerance (set to 15 mm in this study). Pixels with depth values far from the median receive low weight, preserving sharp depth edges. The final depth estimates for the landmark j is the weighted average:

(4) $${\hat{d}}_{j}=\frac{\sum_{k\in N_{j}}w_{k}\cdot d_{k}}{\sum_{k\in N_{j}}w_{k}}$$

F.2 Validity mask

- Reject depth values if:
  ○ Z ∉ [zmin, zmax] = [0.3, 5.0] m, or sensor marks invalid (0), or local dropout > 20%.
- If >20% in a 200 ms
- window are invalid at a landmark, flag for interpolation (Section H).

G. 3D reconstruction (camera → world)

G.1 Back-projection

Given intrinsics K and pixel (u, v) with depth d (meters), back-project to camera frame XC. Then use extrinsic (R, t) to world: XW = RXC + t

G.2 Outlier guardrails

- If landmark jumps > 0.15 m between frames (physical plausibility), mark as transient and send to Hampel filter (Section H)

H. Temporal cleaning and filtering (3D joints and CoM)

- Temporal cleaning is performed in three stages: gaps of 200 ms or fewer (up to 6 frames at 30 Hz) are filled using cubic interpolation; a zero-phase 4th-order Butterworth low-pass filter with a cutoff frequency of 5 Hz is applied to all joint and CoM trajectories; and outliers are suppressed using a Hampel filter (SciPy v1.17.1, SciPy Developers, Austin, TX, USA) with a 0.5 s window and a 3 σ threshold.

I. Ground plane estimation and projection

I.1 Plane estimation

- Region of interest: rectangle spanning both feet using ankle/heel landmarks ± [40–80] px.
- RANSAC plane fit Π: n⊤x + b = 0 on depth points in ROI. Inlier threshold: ε = 8–12 mm distance to plane. Refine using foot landmarks to lock the plane normal.

I.2 Projection

- For any 3D point x project to plane:

(5) $$x_{\Pi }=x-\left(n^{⊤}x+b\right)/{\left\|n\right\|}^{2}n$$

- Stabilogram trajectory is CoM projected to plane: c(t) = [x (t), y (t)] ⊤.

J. Segmental model and whole-body CoM

J.1 Segment definitions

- The body is modeled as 16 rigid segments following the Zatsiorsky–Seluyanov model with de Leva’s adjusted mass fractions [20]. Each segment is defined by a pair of MediaPipe Pose landmarks that serve as proximal and distal endpoints: head (nose to mid-ear midpoint), upper trunk (left shoulder to right shoulder midpoint, to mid-hip midpoint), lower trunk (mid-hip to mid-hip), upper arms (shoulder to elbow), forearms (elbow to wrist), hands (wrist to index finger tip), thighs (hip to knee), shanks (knee to ankle), and feet (ankle to foot index). The segmental center of mass for each segment is located as a fixed proportion along the proximal-to-distal axis, using the gender-specific percentages provided by de Leva [20]. When a landmark is missing due to occlusion or depth dropout, the segment’s position is held at its last valid value for up to 200 ms; beyond that, cubic interpolation is applied as described in Section H. The whole-body CoM is then computed as the mass-weighted sum of all segmental centers of mass using Equation (6).

J.2 Whole-body CoM

(6) $$\mathrm{CoM}\left(t\right)=\sum_{s=1}^{S}w_{s}\;r_{s}\left(t\right),\;\sum w_{s}=1$$

K. Stabilogram feature computation (discrete time)

Let i = 1…N index samples over duration T (fs = 30 Hz) and ci = [xi,yi] ⊤.

1. Path length (PL)

(7) $$PL=\sum_{i=2}^{N}\left\|c_{i}-c_{i-1}\right\|$$

2. Mean Velocity (MV)

(8) $$MV=\frac{PL}{T}$$

3. Directional RMS sway

(9) $$RMS_{AP}=\sqrt{\frac{1}{N}\sum {\left(x_{i}-\bar{x}\right)}^{2}},\;RMS_{ML}=\sqrt{\frac{1}{N}\sum {\left(y_{i}-\bar{y}\right)}^{2}}$$

where x_i_ and y_i_ are the anteroposterior and mediolateral CoM coordinates at sample i, respectively, $\bar{x}$ is the mean AP coordinate over the trial, $\bar{y}$ is the mean ML coordinate over the trial, and N is the total number of samples.

4. 95% ellipse area (EA) (from covariance Σ of x, y)

(10) $$EA=2\pi \;k\sqrt{\lambda_{1}\lambda_{2}},\;k=\sqrt{\chi_{2}^{2}}\approx 2.448$$

where λ_1_ and λ_2_ are the eigenvalues of the 2 × 2 covariance matrix Σ of the projected CoM coordinates (x, y), and $\chi_{2}^{2}$ denotes the chi-squared critical value with 2 degrees of freedom at the 95% confidence level ($\chi_{2}^{2}\approx$ 5.991, giving k = √5.991 ≈ 2.448).

5. Trunk inclination (θ)

- Pelvis → thorax vector v in world frame; vertical unit $\hat{y}$.

(11) $$\theta =\cos^{-1}\left(\frac{v\cdot \hat{y}}{\left|v\right|}\right)$$

L. Quality control (QC) gates and trial inclusion

- Trials are included in the analysis if they meet the following quality criteria: landmark coverage of at least 85% (defined as the proportion of frames with valid pose and depth at key landmarks), per-landmark depth dropout of no more than 20% within the trial, no continuous tracking loss of 5 or more frames (167 ms or more), the subject is positioned within the linear depth region (approximately 1.0–2.5 m), and an investigator is within arm’s reach during quiet stance for safety.

M. Real-time UI and clinician feedback

- Panel A (RGB): 3D skeleton overlay + line of gravity + CoM trace, updated every frame.
- Panel B (Depth heat map): depth normalized to [0, 1]; perceptual colormap; warmer = nearer.
- Panel C (Stick figure): stabilized 3D skeleton in world axes to visualize AP/ML sway and trunk θ.
- Panel D (Metrics): PL, MV, EA, RMS-AP/ML, θ with rolling means; color-code if exceeding thresholds.
- Update cadence: render at display vsync (60 Hz) with last computed metrics.

N. Logging, file schema, and reproducibility

- Per-frame_CSVcolumns: t_capture_ms, t_pose_ms, subj_id, cam_id, joint_0_x… joint_32_z, com_x, com_y, com_z, com_proj_x, com_proj_y, pl, mv, rms_ap, rms_ml, ea, trunk_theta
- Trial JSON sidecar (metadata): camera extrinsics, plane normal n, b, filter settings (fc, order), thresholds, app version hash, OS, GPU driver, room notes.
- Compression: CSV.gz + JSON; hash both files for integrity.

O. Failure modes and defensive coding

- Pose fail: if the global pose detection score s<0.5 for more than 500 ms, freeze GUI metrics, show “tracking lost,” keep logging QC flags.
- Depth voids: if local dropout > 20%, switch to temporal hold-last for that landmark for ≤200 ms; beyond that, interpolate.
- Occlusion: if foot landmarks are unseen, inflate ground plane inlier ε temporarily to avoid plane flips; revert when feet return.

P. Performance tuning

- Threading: 3 threads → (T1) capture, (T2) pose + depth fusion, (T3) filters + metrics + render; lock-free ring buffers.
- Batching: Run pose every frame; if CPU-bound, decimate to 20 Hz and interpolate 2D landmarks back to 30 Hz.
- Profiling hooks: per-stage timers: capture, pose, fusion, filters, metrics, render; export percentile latencies.

**Figure 1:**
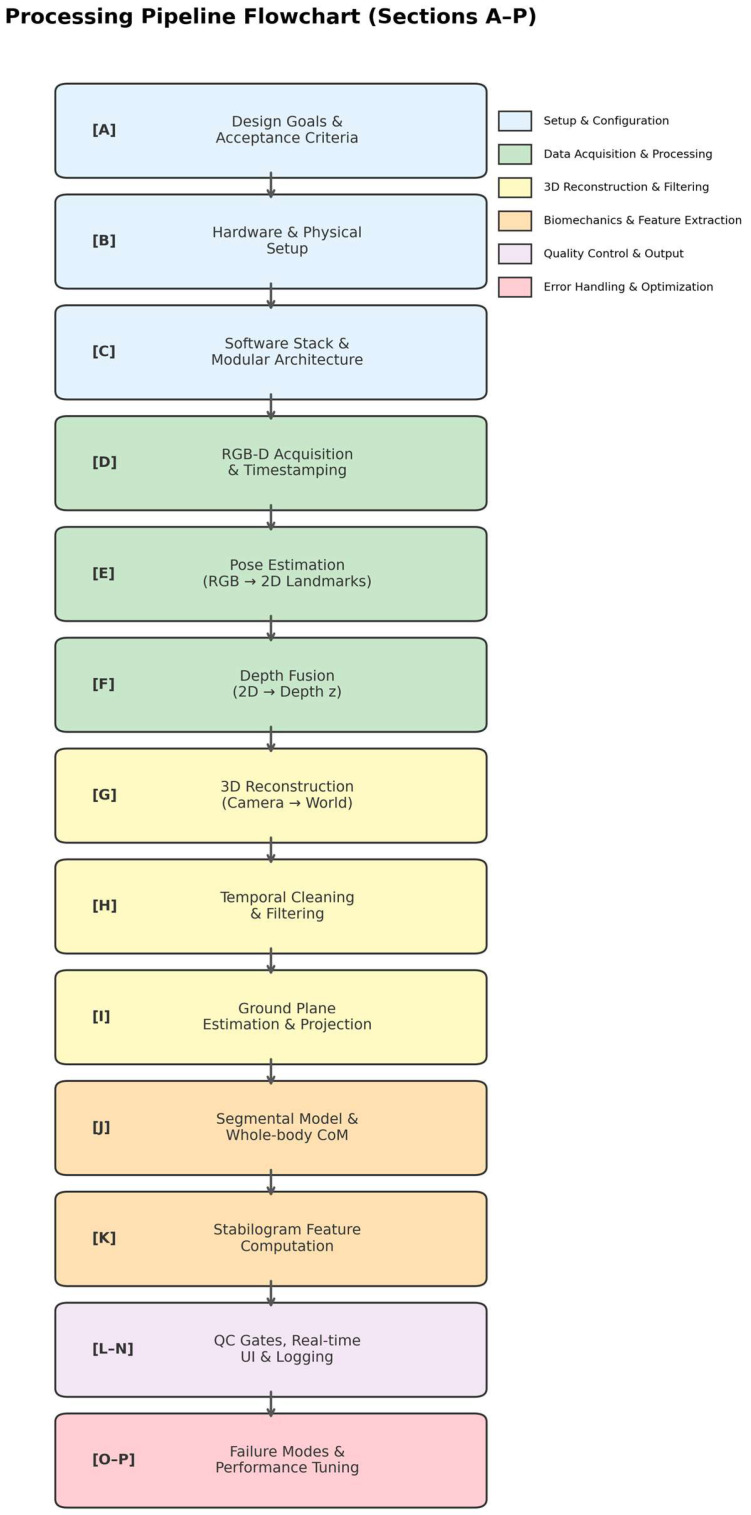
Illustrates the unified algorithmic flowchart of the complete pipeline, integrating all processing stages (Sections A–P) into a single end-to-end overview.

Description of the System Hardware and Software:

We designed a markerless, real-time pipeline to quantify postural sway using a single Intel RealSense D415 depth camera (Figure 1). The system integrates RGB–depth acquisition, skeletal pose estimation, 3D joint reconstruction, biomechanical CoM modeling, and stabilogram feature extraction into a synchronized framework. The workflow proceeds through twelve systematic stages:

6. Hardware Setup: positioning and calibration of the Intel RealSense D415 [17,18].
7. Camera Calibration: intrinsic and extrinsic parameter alignment for accurate 3D reconstruction.
8. Synchronized RGB–Depth Acquisition: recording at 30 Hz, providing millimeter-level precision.
9. Pose and Landmark Estimation: application of MediaPipe Pose, which identifies 33 skeletal landmarks in real time [19].
10. Depth Fusion and 3D Joint Reconstruction: back-projection of pixels into 3D space using intrinsic camera parameters.
11. Ground Plane Estimation: fitting the support plane via RANSAC on foot-depth points and projecting CoM trajectories.
12. Segmental CoM Modeling: calculation of segment-level CoM using the Zatsiorsky–Seluyanov model with adjustments by de Leva [20].
13. Stabilogram Feature Extraction: derivation of sway metrics, including path length (PL), mean velocity (MV), root mean square sway in AP and ML directions (RMS-AP, RMS-ML), ellipse area (EA), and trunk inclination.
14. Data Cleaning and Filtering: signal interpolation, Butterworth low-pass filtering, and artifact suppression.
15. GUI and Heat Map Visualization: generation of a four-panel display, including RGB with skeleton overlay, depth heat map, 3D stick figure, and live stabilogram metrics.
16. Logging and Synchronization: continuous logging of joint and CoM time-series data.
17. Statistical and Error Analysis: evaluation of reliability (ICC) and validity (correlation, Bland–Altman analysis [48]) against Mocap reference values.

This structured approach ensures that data acquisition, processing, and validation follows a reproducible pipeline, enabling rigorous comparison of RealSense-derived sway metrics with laboratory-grade standards.

Data Quality, Inclusion, and Safety

Trials are retained if: landmark coverage ≥ 85%, depth dropout ≤ 20%, and no tracking loss ≥ 5 consecutive frames. Participants stand near the center of the force plate (validity phase) and within the depth linear region (1.0–2.5 m). Investigators remain within arm’s reach for safety.

GUI, Heat Map, and Four-Panel Composer

**Figure 2:**
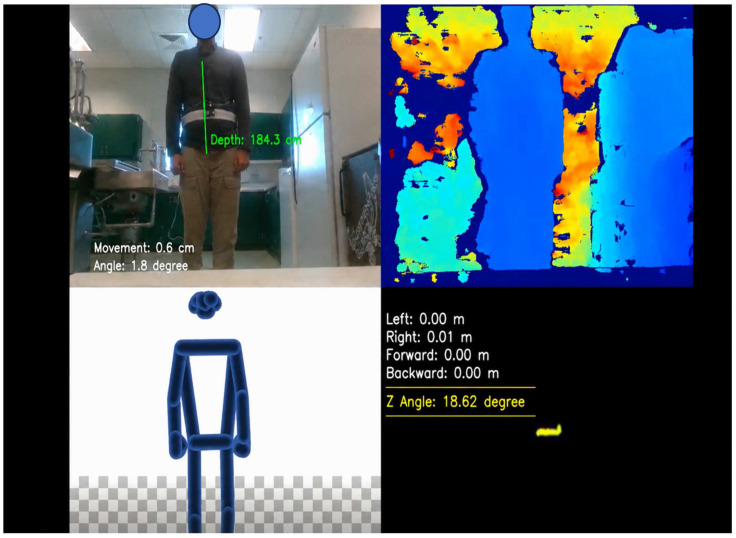

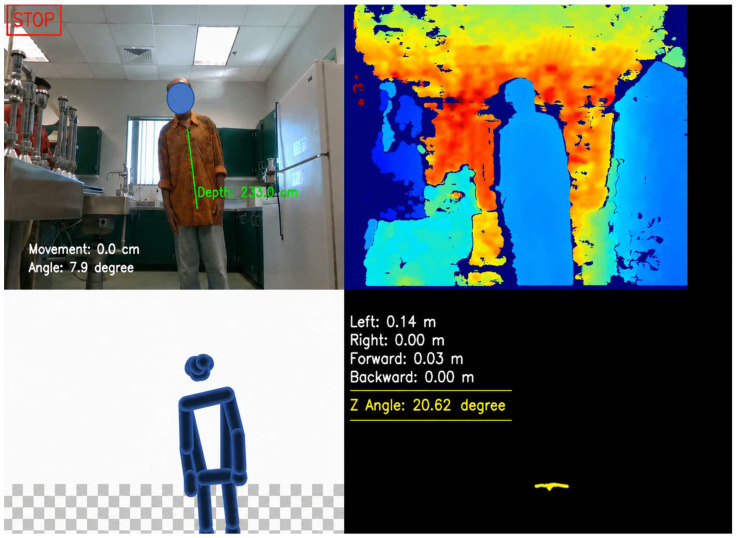
A four-panel graphical user interface for the user.

We render a real-time quad-view for clinician feedback (Figure 2):

- Panel A (RGB): overlaid 3D skeleton and line of gravity; CoM trajectory trace.
- Panel B (Depth heat map): depth normalized to [0, 1] and mapped to a perceptual colormap; warmer colors = nearer.
- Panel C (Stick figure): stabilized 3D skeleton in world axes to visualize AP/ML sway and trunk inclination.
- Panel D (Metrics): PL, MV, EA, RMS-AP/ML, *θZ* updated each frame with rolling means.

Logging and Synchronization

The information that was logged per-frame was timestamps, 3D joints, CoM (*x*, *y*, *z*), projected (*x*, *y*), and all metrics were exported to CSV format.

Phase II—Experimental Testing:

The experimental study was designed to evaluate the test–retest reliability and concurrent validity of the RealSense D415-based 3D camera system for quantifying postural sway and balance. The study was reviewed and approved by the ethics committee of Florida Atlantic University, and all subjects signed an informed consent prior to study participation. A total of 10 healthy university students (6 males, 4 females; mean age 22.3 ± 1.6 years, mean height 168.4 ± 8.7 cm, mean body mass 67.2 ± 10.4 kg) were recruited to participate in the study. Inclusion criteria required that participants be aged 18 years or older and have no self-reported history of balance disorders, neurological conditions, or lower-extremity injuries within the past six months.

Data were collected simultaneously from the Mocap system (Optotrak, NDI Digital, Waterloo, ON, Canada) and the 3D camera. The 3D camera was connected to a Lenovo laptop computer and was placed on a flat surface below the Optotrak camera, which was mounted on a stand. For Mocap data collection, a single marker was placed on the subjects’ waist at the midline, using a waist belt. Participants wore form-fitting clothing to minimize depth-sensor occlusion from loose fabric, and testing was performed barefoot on a flat, non-slip surface. Stance width was standardized by instructing participants to place their feet hip-width apart, with foot placement marks on the floor to ensure consistent positioning across trials and sessions. A visual fixation target was placed on the wall at eye level, approximately 2 m in front of the participant, to standardize gaze direction. Testing was conducted under uniform indoor fluorescent lighting with no direct sunlight or strong infrared sources in the room. The camera-to-subject distance was fixed at 2.0 m for all trials. All testing sessions were conducted by the same investigator to eliminate inter-rater variability, and environmental conditions (room, lighting, camera position, time of day) were reproduced identically during the second assessment session performed 48–72 h later. Before data collection, each participant received standardized verbal instructions describing the four movement directions and performed one practice trial for each direction to familiarize themselves with the task. To calculate CoM path length, subjects performed the following movements: forward lean (without moving the feet), backward lean, left lean, and right lean. When subjects reached their farthest position, they were asked to hold that position for 3 s. Each movement was repeated 3 times, and data were collected for 20 s at a frequency of 30 Hz from both systems simultaneously. Within each testing session, approximately 30 s of rest was provided between consecutive trials of the same movement. The maximum path length across the three repetitions was used for analysis. For test–retest reliability, participants returned for a second session within 48–72 h, performed under identical conditions (same room, same camera position, same time of day).

It is important to distinguish between two levels of synchronization in this study. Within the RealSense system, the RGB and depth streams are hardware-synchronized by the camera’s internal clock and delivered as aligned frame pairs at 30 Hz; there is no temporal offset between the color image used for pose estimation and the depth map used for 3D reconstruction. However, between the RealSense and Optotrak systems, no electronic synchronization (e.g., a shared trigger or common clock) was implemented. This was a deliberate design choice: because the variable of interest was maximum path length over each 20-s trial rather than instantaneous frame-by-frame trajectory agreement, precise temporal alignment between the two systems was not required. Each system independently recorded its own complete trial, and the maximum path length was extracted from each recording separately. No post-processing temporal alignment or frame-matching procedures were applied.

Data Analysis: To determine test–retest reliability, ICC values were calculated for the path lengths obtained during the two testing sessions. ICCs were calculated for each of the four subject movements, i.e., forward, backward, left, and right movements. Concurrent validity was determined by comparing the path lengths obtained with the 3D camera with those obtained from the Optotrak system. Pearson’s correlation coefficient, the accuracy of length measurement between the two systems, and the Bland–Altman plot of agreement were used to assess concurrent validity [1,48].

## 3. Results

**Figure 3:**
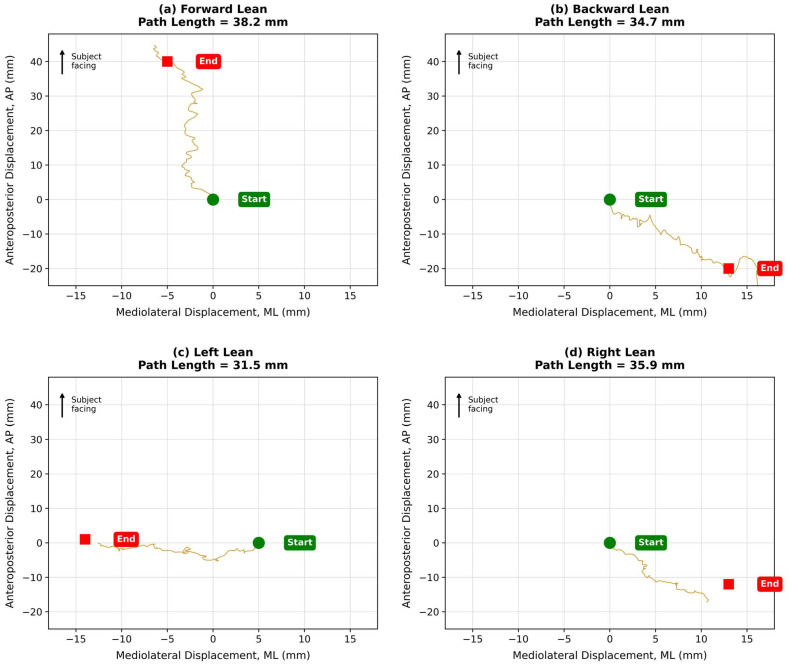
Stabilograms generated for a representative subject during (**a**) forward lean, (**b**) backward lean, (**c**) left lean, and (**d**) right lean. Axes indicate mediolateral (ML) and anteroposterior (AP) displacement of the projected center of mass in millimeters. The arrow indicates the subject’s facing direction.

Figure 3 shows the anterior–posterior and medial–lateral movements of the CoM, which were captured with the 3D camera system. The numbers displayed on the figure represent the path lengths. In terms of the Phase II experimental testing, the ICCs were 0.86 (95% CI: 0.63–0.96), 0.84 (95% CI: 0.58–0.95), 0.86 (95% CI: 0.63–0.96), and 0.85 (95% CI: 0.60–0.95) for forward, backward, right, and left movements, respectively. The ICC model used: two-way mixed, absolute agreement, single measures. The error between the 3D camera and Optotrak was 10.5% on average. Pearson’s correlation coefficient between the two systems averaged 0.8, indicating high correlation in path length measurements by the two systems.

**Figure 4:**
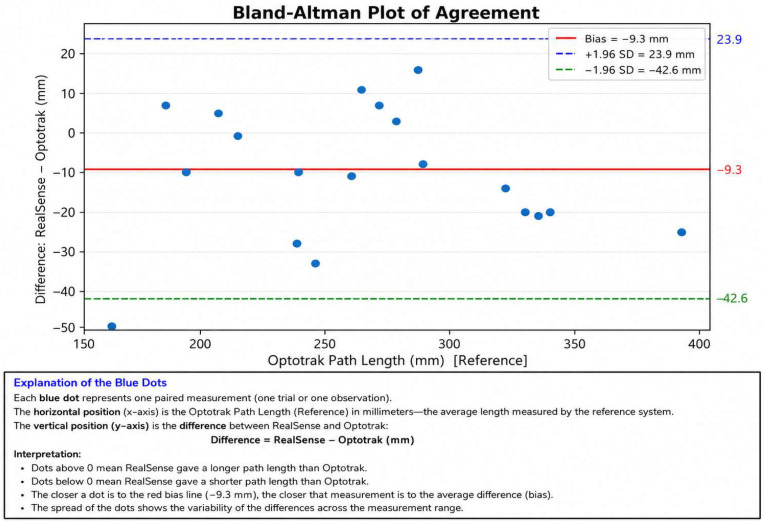
Bland–Altman plot of agreement between RealSense and Optotrak path length measurements (mm). The horizontal axis represents the Optotrak reference measurement. The vertical axis represents the difference (RealSense minus Optotrak). The solid red line indicates the mean difference (bias = −9.3 mm), and the dashed lines indicate the 95% limits of agreement (+23.9 mm to −42.6 mm).

The level of agreement between the two systems was determined using a Bland–Altman plot (Figure 4). This figure is for the data when the subject moved to their right side and is representative of the data for the other movements as well.

All 120 trials (10 participants × 4 directions × 3 repetitions) met the quality control inclusion criteria (landmark coverage ≥ 85%, depth dropout ≤ 20%, no tracking loss ≥ 5 consecutive frames); no trials were excluded from the analysis.

The Bland–Altman plot showed a good agreement between the 3D camera system and the Optotrak system. The mean difference (bias) between the RealSense and Optotrak systems was −6.6 mm, indicating that the 3D camera produced a slight systematic underestimation of path length relative to the Optotrak reference. The standard deviation of the differences was 15.1 mm. The 95% limits of agreement ranged from −36.1 to 22.9 mm. These values confirm that the systematic error is small relative to the measured path lengths, and the variability between the two systems is within clinically acceptable limits.

The 95% limits of agreement (−42.6 to +23.9 mm) are relatively wide compared to the measured path lengths (range approximately 140–380 mm), representing approximately 17–30% of the measurement range. This indicates that individual-level interchangeability between systems cannot be assumed, and the RealSense system should currently be considered a screening tool rather than a replacement for laboratory-grade systems.

While the Pearson correlation (r = 0.80) reflects a strong linear association between systems, it does not indicate agreement; the Bland–Altman analysis is therefore the primary measure of concurrent validity.

## 4. Discussion

The experimental study evaluated the reliability and validity of the RealSense D415-based 3D camera system for quantifying postural sway and balance. Our findings indicate that the system is reliable and valid among young, healthy individuals and has clinically acceptable accuracy. Results suggest that the system is sensitive enough to detect clinically meaningful changes in sway and is reliable for repeated use, confirming its potential as a clinically viable alternative for balance assessment. These findings are consistent with recent depth camera validation studies [35,48] and surpass results from earlier Kinect-based systems.

Unlike traditional 2D video or subjective clinician observation, 3D cameras capture multiplanar sway (anteroposterior, mediolateral, and vertical) as well as trunk inclination, providing a more complete profile of postural control. Integration with biomechanical models, such as the Zatsiorsky–Seluyanov framework with de Leva’s refinements, allows sway trajectories to be interpreted in terms of whole-body center-of-mass dynamics rather than isolated joint motion [20]. Furthermore, the system’s ability to generate real-time stabilograms and heat maps supports immediate clinical feedback. This is particularly useful in emergency and rehabilitation contexts, where rapid identification of subtle sway abnormalities can guide decision-making. Wearable inertial sensors have demonstrated reliable and valid postural sway measurements across progressively challenging balance tasks including unstable surfaces, further supporting portable sensor-based balance assessment [49]. These findings align with recent evidence showing that inertial sensors maintain reliable and valid sway measurements even across five progressively unstable surface conditions, reinforcing the broader applicability of portable balance assessment tools [49].

The 3D camera system compares favorably with other portable systems for balance and sway assessment in this preliminary evaluation. While IMUs provide portability and moderate reliability, they only provide indirect estimates of CoM. They are also sensitive to placement errors and drift [13], which could reduce the consistency of measurement between clinicians. In contrast, RealSense offers direct 3D reconstruction without wearable devices. The other low-cost pressure plates, such as the Wii Balance Board and BtrackS, measure CoM with acceptable repeatability [32], but lack a full-body kinematic context. The Kinect camera system pioneered the markerless balance evaluation [35,36], but limitations in depth accuracy, resolution, and occlusion restricted its clinical application [12,35]. RealSense D415 addresses these shortcomings with sub-millimeter depth precision, robust skeletal tracking, and improved reliability [17,18].

Several methodological factors may have contributed to the observed disagreement between the RealSense and Optotrak systems. Most notably, the two systems employ fundamentally different biomechanical approaches: the RealSense system estimates whole-body center-of-mass trajectories from 33 markerless skeletal landmarks using a 16-segment biomechanical model, whereas the Optotrak setup tracked a single reflective marker placed at the waist midline. The single-marker approach approximates CoM displacement but does not account for the contributions of upper-limb, head, or lower-limb segment movements, which may lead to systematic differences in path length estimation, particularly during directional leaning tasks where trunk and limb contributions diverge. Additionally, differences in spatial precision between the two sensors may have influenced the results: the Optotrak system provides sub-millimeter accuracy for marker tracking, whereas the RealSense D415 has depth noise on the order of 1–2 mm at 2 m range [17], and the MediaPipe Pose landmark localization introduces additional 2D positioning uncertainty. The low-pass filtering parameters also differed between systems (the RealSense pipeline applied a 5 Hz Butterworth filter, whereas the Optotrak data were processed using the manufacturer’s default settings), which may affect the smoothness and total path length of the resulting trajectories. Finally, because the two systems were not electronically synchronized, small differences in trial start and end times may have produced slightly different maximum path length values, though this effect is expected to be minimal given the 20-s trial duration and the use of maximum rather than instantaneous values.

The findings of this study are consistent with the growing body of literature demonstrating that portable, non-laboratory-based systems can provide reliable and valid balance assessments [5,14,21], further supporting the clinical applicability of the proposed 3D camera system. These results are further supported by validation studies confirming that whole-body center-of-mass sway can be accurately estimated using multi-segment IMU models with errors below 1 mm in both AP and ML directions during quiet standing [50].

Clinical Implications:

The affordability (~USD 200), portability, and real-time capabilities of the RealSense system make it suitable for broad deployment beyond laboratories, including outpatient clinics, rehabilitation centers, community health programs, and tele-health platforms. A portable 3D camera-based support system could enable clinicians to detect abnormal sway patterns within minutes, facilitating triage decisions, monitoring deterioration or recovery, and guiding timely interventions to reduce fall risk during hospitalization [2]. Sensor-based interventions using wearable and camera-based technologies have demonstrated significant improvements in postural balance outcomes in older adults [25,30], reinforcing the translational potential of the proposed system for real-world balance rehabilitation. Markerless motion capture apps have been validated for automated scoring of clinical functional tests, including the Timed Up and Go, in adults with chronic disease [40]. Moreover, sway analysis can complement existing emergency protocols by providing objective quantitative data to support clinical judgment, particularly in cases where visual observation alone may underestimate subtle balance deficits. In the rehabilitation of older adults and neurological patients, the system can provide objective metrics to track progress and offer rapid and non-invasive insights into acute neurological or vestibular dysfunction. It could also serve as a low-cost fall risk screening tool in clinics. This combination of portability, immediacy, and objectivity makes 3D sway estimation a promising tool for integration into acute neurological care and rehabilitation clinics.

The accuracy of the markerless pose estimation may be affected by several factors not systematically controlled in this study. Loose or baggy clothing can occlude joint landmarks, potentially increase depth dropout rates, and reduce pose estimation confidence. Clothing colors that closely match the background may reduce contrast in the RGB stream, affecting landmark detection. Body dimensions, particularly limb proportions and body mass index, influence the visibility and spacing of skeletal landmarks; the system was evaluated only on young, healthy adults of typical build, and performance on individuals with atypical body proportions remains to be validated. Ambient lighting conditions and the presence of strong infrared sources near the camera may also affect depth map quality.

In real clinical settings, additional challenges may arise that were not present in the current controlled evaluation. Patients using assistive devices such as walkers, canes, or wheelchairs may partially occlude lower-limb landmarks, reducing pose estimation accuracy. Individuals with neurological disorders such as Parkinson’s disease, stroke, or vestibular dysfunction frequently exhibit compensatory movement patterns, tremor, trunk flexion, and asymmetrical posture, which may challenge the assumptions of the MediaPipe Pose model and affect the reliability of CoM estimation. Similarly, patients with severe balance impairments may require physical support from an investigator, introducing an additional body into the camera’s field of view and potentially interfering with skeletal tracking.

These factors should be investigated in future work.

## 5. Limitations

This study has several limitations. First, the sample consisted of young, healthy university students, which limit generalizability to older adults or clinical populations with neurological or musculoskeletal conditions. Future validation studies should include older adults and clinical populations, as balance deficits and fall risk are most prevalent in these groups [4,25]. Markerless motion capture has shown particular promise for quantifying functional performance in populations with neurodegenerative conditions [6], and such validation would significantly strengthen the clinical relevance of the proposed system. Second, only quiet-standing lean tasks were evaluated; the system’s performance during dynamic balance tasks such as gait initiation or perturbation responses has not been assessed. Third, the RealSense D415 operates at 30 Hz, which may be insufficient for capturing rapid postural adjustments that higher-frequency force platforms (e.g., 200 Hz) can detect. Fourth, the two measurement systems were not electronically synchronized; only the maximum path length was compared, and time-series agreement was not evaluated. Fifth, all testing was performed under controlled indoor lighting, and system performance under variable lighting conditions has not been tested. Finally, the influence of clothing type, body dimensions, and skin tone on pose estimation accuracy was not systematically evaluated in this study. Additionally, the Zatsiorsky–Seluyanov model applies fixed population-averaged mass fractions, which may not accurately represent individuals with atypical body composition or proportions. Future iterations could incorporate subject-specific anthropometric parameters—such as body volume, height, and weight estimated directly from 3D camera data [51,52] to refine segmental mass modeling.

## 6. Future Work

The findings of the experimental study highlight several important directions for extending the capabilities and applications of 3D camera-based sway assessment:

1. Validation in Clinical Populations: This study focused on healthy young adults, which limits generalizability to older or clinical populations. Future studies should include older adults, patients with Parkinson’s disease, vestibular dysfunction, and stroke survivors to establish generalizability. Testing across diverse cohorts will confirm whether RealSense-based metrics remain reliable and valid in populations most at risk of falls.
2. Dynamic Balance Tasks: The current evaluation only evaluated quiet-standing tasks. Beyond quiet standing, protocols such as the Modified Clinical Test of Sensory Interaction on Balance, tandem stance, sit-to-stand, gait initiation, and perturbation tasks should be systematically evaluated [53].
3. Multi-Camera Fusion: Incorporating multiple synchronized RealSense devices in convergent positions can minimize occlusion errors, expand field-of-view coverage, and improve depth linearity. Multi-camera setups may also enable more accurate 3D reconstructions of whole-body kinematics during dynamic tasks.
4. Machine Learning and Predictive Analytics: Integration of advanced machine learning algorithms can enable automated classification of high-risk vs. low-risk individuals based on sway features. Machine learning approaches for fall risk classification have already been demonstrated using wearable plantar pressure sensors [33] and markerless motion capture systems [54], providing a clear pathway for incorporating predictive analytics into the proposed 3D camera system. The results suggest that such low-cost, portable approaches could support predictive models for fall risk and promote personalized rehabilitation strategies. Machine learning applied to triaxial inertial sensor data has shown promise for predicting center-of-pressure trajectory during postural sway, supporting the development of automated balance assessment tools [55].
5. Standardizing testing protocols: Development of standardized testing protocols using different commercially available cameras, as well as the creation of normative databases for healthy individuals and those with specific conditions, such as vertigo, will be beneficial for translating this technology into clinical practice.

## 7. Conclusions

A novel 3D camera-based postural sway and balance measurement system has been presented along with the algorithmic pipeline to quantify postural sway in real time. The system performs skeletal landmark detection along with real-time RGB–depth acquisition. It accurately estimates the segmental CoM through the Zatsiorsky–Seluyanov model. Based on preliminary testing with healthy adults, the system shows promise as a low-cost alternative to lab-based motion capture systems. Further validation in clinical populations is needed before it can be recommended for clinical assessment, tele-rehabilitation, or large-scale fall risk screening.

The experimental study demonstrated that an Intel RealSense D415-based 3D camera system can provide reliable and valid quantification of postural sway and balance during quiet standing. By combining synchronized RGB–depth acquisition, real-time skeletal landmark detection, and biomechanical center-of-mass modeling, the system achieved excellent test–retest reliability (ICC = 0.84–0.86), good concurrent validity, and a high level of agreement when compared with a laboratory-based system.

The results suggest that low-cost, portable, and markerless depth camera technology has the potential to approximate laboratory-level sway metrics, though further validation across diverse populations and dynamic tasks is required to confirm clinical-grade precision. These findings are consistent with recent literature confirming that portable sensor-based systems—both IMU and camera-based—can reliably quantify balance and sway metrics comparable to laboratory gold standards [14,15,21,30], highlighting the broader translational potential of accessible balance assessment technology. Unlike traditional motion capture systems, the RealSense system is affordable, easy to deploy, and capable of providing immediate visual feedback through stabilograms and heat maps. These features make it well-suited for diverse environments, including outpatient clinics, rehabilitation centers, tele-health platforms, community health programs, and even emergency care settings where rapid screening may guide decision-making.

By bridging the gap between high-fidelity laboratory instrumentation and accessible point-of-care solutions, this work lays the foundation for the wider adoption of 3D camera-based balance assessment. With further validation in clinical populations and dynamic tasks, integration of multi-camera setups, and incorporation of machine learning analytics, such systems may eventually support fall risk screening, rehabilitation monitoring, and personalized balance interventions, pending further clinical validation.

## Data Availability

The original contributions presented in this study are included in the article. Further inquiries can be directed to the corresponding author.
